# Stochastic models of Mendelian and reverse transcriptional inheritance in state-structured cancer populations

**DOI:** 10.1038/s41598-022-17456-w

**Published:** 2022-07-29

**Authors:** Anuraag Bukkuri, Kenneth J. Pienta, Robert H. Austin, Emma U. Hammarlund, Sarah R. Amend, Joel S. Brown

**Affiliations:** 1 Cancer Biology and Evolution Program and Department of Integrated Mathematical Oncology, Moffitt Cancer Center, Tampa, USA; 2 The Brady Urological Institute, Johns Hopkins School of Medicine, Baltimore, USA; 3 Department of Physics, Princeton University, Princeton, USA; 4 Nordic Center for Earth Evolution, University of Southern Denmark and Translational Cancer Research, Department of Laboratory Medicine, Lund University, Lund, Sweden

**Keywords:** Ecological modelling, Population dynamics, Evolutionary ecology, Theoretical ecology, Evolutionary theory, Applied mathematics

## Abstract

Recent evidence suggests that a polyaneuploid cancer cell (PACC) state may play a key role in the adaptation of cancer cells to stressful environments and in promoting therapeutic resistance. The PACC state allows cancer cells to pause cell division and to avoid DNA damage and programmed cell death. Transition to the PACC state may also lead to an increase in the cancer cell’s ability to generate heritable variation (evolvability). One way this can occur is through evolutionary triage. Under this framework, cells gradually gain resistance by scaling hills on a fitness landscape through a process of mutation and selection. Another way this can happen is through self-genetic modification whereby cells in the PACC state find a viable solution to the stressor and then undergo depolyploidization, passing it on to their heritably resistant progeny. Here, we develop a stochastic model to simulate both of these evolutionary frameworks. We examine the impact of treatment dosage and extent of self-genetic modification on eco-evolutionary dynamics of cancer cells with aneuploid and PACC states. We find that under low doses of therapy, evolutionary triage performs better whereas under high doses of therapy, self-genetic modification is favored. This study generates predictions for teasing apart these biological hypotheses, examines the implications of each in the context of cancer, and provides a modeling framework to compare Mendelian and non-traditional forms of inheritance.

## Introduction

Despite major advances in cancer research, advanced cancers largely remain incurable due to the evolution of resistance, killing more than 10 million people per year^1,2^. Traditionally, it is assumed that cancers randomly mutate in the presence of therapy^3^. Some of these stochastic mutations confer a fitness advantage to cells by making them more resistant to the therapy. These cells then go on to proliferate at higher rates in the population. In this way, the cancer becomes increasingly resistant to therapy, eventually rendering the therapy ineffective^4^. However, recent experimental evidence indicates another key factor in the emergence of resistance: the polyaneuploid cancer cell (PACC) state^1,5–7^. Cancer cells in the aneuploid 2N+ state can enter the PACC state by bypassing mitosis, cyotkinesis, or both and resulting in a whole genome doubling^5,7,8^. The non-proliferative PACC cell state is characterized by two distinct features: 1) an exit of the cell cycle after S phase to pause division and allow cancer cells to withstand stressful conditions^9^ and 2) an increase in genomic material due to polyploidization which is assumed to contribute to a greater level of heritable variation (evolvability)^10–14^ that can be passed on to their progeny when they undergo depolyploidization into the 2N+ state^15–18^. In this way, the PACC state provides a mechanism for *facultative evolvability* in the cancer cell population in which cells can increase their evolvability (and thus, their rate of adaptation) during times of stress and decrease it during times of stability.

The ecological and evolutionary dynamics underlying these processes remain an enigma. Under classical notions of Mendelian inheritance and evolutionary triage (ET)^19^, organisms scale adaptive landscapes in a process of creative destruction. Due to the struggle for existence, this inherently requires the proliferation of more fit 2N+ cells and the death of less fit 2N+ cells in the population (in the context of therapy, this leads to the death of more sensitive cells and the proliferation of more resistant ones)^20^. However, observations of cancer cell population dynamics with 2N+ and PACC states under therapy cannot be reconciled solely with this theory. Under extreme stress, we notice a drastic transition of cells from the 2N+ state to the non-proliferative PACC state^5,21–26^. After a delay, cells in the PACC state begin depolyploidizing into 2N+ progeny. Critically, these progeny immediately display high levels of resistance to the stressor. Unlike as would be expected under ET, we do not observe the death of mutant clones. In other words, this evolutionary process appears to “solve the therapeutic problem” without mortality of less successful variants relative to more successful ones^27,28^.

One way this can occur is through the process of self (epi-)genetic modification, hereafter referred to as self-genetic modification (SGM)^29–31^. SGM, also described as genetic assimilation, was originally described by Waddington as an interplay between traits acquired during an organism’s lifetime and the genetic encoding of these traits^32,33^. In Lamarckian fashion, reverse transcription allows changes that a cell undergoes during its own lifetime to be written back into its DNA and passed onto its offspring^34–36^. This process was first identified in the context of the immune system and was proposed as an explanation for why homologous DNA sequences from VDJ gene regions of parent mice are found in their germ cells and were passed on to offspring for subsequent generations^36–38^. The concept of reverse transcriptional inheritance is closely related to the idea of adaptive or directed mutations, a process in which mutations are produced to relieve the selective pressure^39^ in that death of less fit variants is not required for evolution to proceed.

In the context of cancer, under stress, cells in the PACC state may be able to assess their lack of viability, devise a viable solution, and code this back into their DNA^30,31,40^. These adaptations are heritable and are thus directly passed on to their 2N+ progeny upon depolyploidization. Evolution in this sense does not require death of 2N+ cells: rather than climbing hills on their adaptive landscape due to mutation and selection, cells under SGM “jump” up the landscape, displaying short bursts of rapid evolutionary change. Such processes are not unique to PACCs; similar phenomena have also been observed in yeast^41^, *Drosophila*^32,42–44^, invasive herbs^45,46^, butterflies^47^, tiger snakes^48^, *Anolis* lizards^49,50^, grasshoppers^51,52^, yarrow^53–56^, and bacteria^39,57,58^, in which a variety of stressors such as nutrient depletion, change in temperature, DNA damage, and exposure to antibiotics can induce mutagenesis via global response systems such as activation of DNA polymerases, downregulation of error-correcting enzymes, and movement of mobile genetic elements^57^. Though these mechanisms increase heritable mutation, evidence also supports the selective capture of mutations directed towards the stressors at hand^59–68^.

Despite several theoretical studies on mechanisms of inheritance similar to reverse transcription^69–78^, robust methods do not exist to broadly model coupled ecological and evolutionary dynamics under such inheritance and to compare these results to those under classical Mendelian inheritance. Furthermore, none of these studies investigate reverse transcription in particular and of those that consider Lamarckian inheritance more generally, none do so in the context of cancer. Although we specifically focus on creating stochastic mathematical models of Mendelian and reverse transcriptional inheritance in the context of cancer cell populations with 2N+ and PACC states under therapy, our approach is broadly applicable and can serve as a general modeling framework within which mechanisms of reverse transcriptional and Mendelian inheritance can be explored in a variety of contexts. To construct this model, we build on a model we developed in prior work and outline a birth–death switching process algorithm to simulate the ET and SGM hypotheses stochastically. We examine how the eco-evolutionary dynamics of the cancer cell populations are affected by drug dosage and extent of SGM. Finally, we run *in silico* competition experiments to help us understand whether ET or SGM performs better under soft (low drug dosage) and hard (high drug dosage) selection regimes.

## Model construction

### ODE model of 2N+ and PACC ecology

In order to stochastically simulate the eco-evolutionary dynamics of cancer cell populations with 2N+ and PACC states, we first construct an underlying ODE model, from which we can derive birth, death, and switching rates. To do this, we first create a model of the population dynamics and state transitions for the 2N+ and PACC states in Eq. (1).

1 $$\begin{aligned} \begin{aligned} \frac{dx_1}{dt}&= \underbrace{rx_1\Big (\frac{K-x_1-x_2}{K}\Big )}_\text {Logistic Growth}\overbrace{-\gamma x_1}^\text {Obligate to PACC}\underbrace{-x_1\frac{m}{\lambda +bv}}_\text {Drug-Induced Death}\overbrace{-c_{21}x_1\frac{m}{\lambda +bv}}^\text {Facultative to PACC}\underbrace{+2c_{12}x_2}_\text {From PACC}\\ \frac{dx_2}{dt}&= \underbrace{\gamma \eta x_1}_\text {Obligate from 2N+}\overbrace{+c_{21}\eta x_1\frac{m}{\lambda +bv}}^\text {Facultative from 2N+}\underbrace{-c_{12} x_2}_\text {To 2N+} \end{aligned} \end{aligned}$$

Here, $x_1$ and $x_2$ represent the population of cells in the 2N+ and PACC states respectively and *v* represents a resistance strategy such as the number of drug antiporters, receptor levels, gene expression levels, allocation to different metabolic pathways, or upregulation of DNA repair processes. We make several assumptions: cells in the PACC do not die from therapy, competition, or natural causes (i.e., no background death); our resistance strategy is a continuous quantitative trait; cells within the 2N+ and PACC states are identical and posess the same resistance strategy (though it is not necessary while a cell is in the PACC state); cells in the PACC state are all 4N+ in ploidy (actual measures of PACCs find this and even higher levels of ploidy); and the anueploid cancer cell population is pre-existing.

The 2N+ dynamics emerge from the following. We assume logistic growth, in which cells in the 2N+ state are inhibited equally by cells in the 2N+ and PACC states, until they reach their carrying capacity. We allow for an obligate (constant) transition of cells to the PACC state, an assumption supported by experimental literature that observes a baseline level of PACCs in most cancer cell populations^5^. We let drug induced mortality take on a Michaelis–Menten form, in which mortality is a decreasing function of resistance. We also include a facultative or condition-dependent transition to the PACC state. The higher the death in 2N+ cells due to the drug, the greater their transition rate to the PACC state. These facultative transitions are observed experimentally, with an increase in PACC frequency and number upon administration of therapy^22^. Finally, we imagine a constant transition rate of cells from the PACC state into the 2N+ state. Note that since we assume cells in the PACC state are 4N+ in ploidy, a depolyplodization event produces two 2N+ cells.

The PACC cell dynamics emerge from the following. Since cells in the PACC state cannot divide without depolyploidizing into the 2N+ state, cells in the PACC state have no inherent growth rate. We also assume that cells in the PACC state are fully resistant to therapy. PACC dynamics are solely influenced by obligate and facultative transitions from the 2N+ state and a constant transition back to the 2N+ state. For the former, we also include a successful polyaneuploid transition probability, $\eta$, to account for the possibility of failed polyploidization that leads to cell death, as is sometimes observed experimentally.

Table 1 provides parameter values and their interpretations. All values were chosen to be biologically realistic, numerically convenient, and to clearly show differences between simulation results. Many of these parameter values can qualitatively alter our results, however. Anytime the intrinsic growth rate of the 2N+ cells, *r*, is higher than the maximum potential kill rate of the drug, $m/\lambda$, the cancer will survive therapy even in the absence of evolving resistance. As expected, a higher carrying capacities lead to higher equilibrium population sizes. We present the results of sensitivity analyses of the key parameters in the supplemental material. With this model, we can now outline a method for stochastically stimulating the ET and SGM hypotheses.

**Table 1:** Parameter definitions and values used in simulations.

Parameter	Interpretation	Value
*r*	Intrinsic growth rate	0.6 $\hbox {day}^{-1}$
*K*	Carrying capacity	100 $*10^7$ cells
$\gamma$	Obligate transition rate	0.02 $\hbox {day}^{-1}$
*m*	Drug dosage	Varies $\hbox {day}^{-1}$
$\lambda$	Baseline level of resistance	1
*b*	Efficacy of evolving a resistance strategy	1
$c_{21}$	Facultative transition scaling rate	0.7 $\hbox {day}^{-1}$
$c_{12}$	PACC to 2N+ transition rate	0.2 $\hbox {day}^{-1}$
$\eta$	Successful polyaneuploid transition probability	0.7
$\mu$	Mutation rate	0.05 $\hbox {division}^{-1}$
$\sigma _1$	Breadth of mutation: 2N+ Division	0.01
$\sigma _2$	Breadth of mutation: PACC Depolyploidization	0.05
$\zeta$	Self-genetic modification threshold	Varies $*10^7$ cells $\hbox {day}^{-1}$
*v*	Drug resistance strategy	$[0,\infty )$

### Stochastic simulation: birth–death-switching process

To simulate the eco-evolutionary dynamics of this state-structured cancer cell population, we use a birth–death-switching process. We provide an intuitive explanation of this process that parallels Hammerstein’s streetcar theory of evolution with several temporary stops determined by the underlying genetic architecture and a “final stop” determined by phenotypic selection that promotes ecological stability^79^. The implementation of this process is closely related to Dieckmann’s directed random walks in adaptive dynamics^80–82^ in which evolution proceeds as a sequence of trait substitutions, as mutant traits with positive invasion fitness replace the resident trait in the population^83^. The simulation implements the stochastic element at the population level, tracking changes in trait values and population sizes at different cell state compartments.

**Figure 1:**
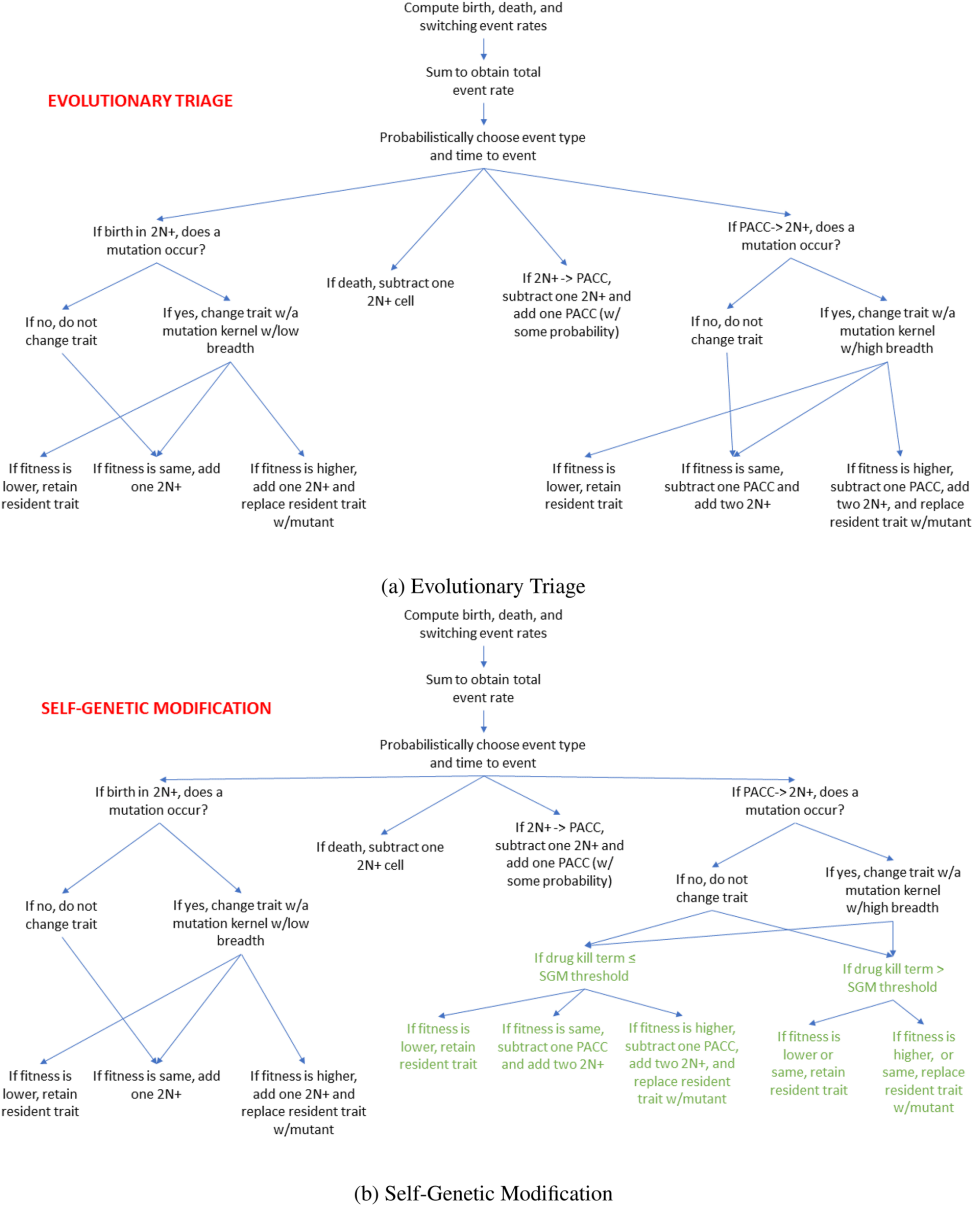
Flowcharts depicting the probabilistic and hence stochastic steps for the simulations of evolutionary triage (**a**) and self-genetic modification (**b**). The green text indicates where in the simulation process SGM differs from ET. See the Supplemental Material for a thorough explanation for how Eq. (1) translates into the stochastic birth–death switching process.

### Evolutionary triage

Under ET, when the cancer cell population is under stress, cells transition from the 2N+ to the PACC state. This state provides a refuge for cells under therapy and, like Goldschmidt’s hopeful monster^84^, produces profound mutant progeny “in hopes” that they will remain viable in the face of therapy. This high mutational breadth is the result of the increased genomic content that accompanies the polyploidization program of cells as they transition into the PACC state. If the mutant progeny has a higher fitness than the residents in the population, it will eventually sweep through the population and replace the resident trait. If the mutant has a lower fitness than the resident, it will be driven to extinction. In this process of creative destruction, the cancer cells will climb an adaptive landscape via the mortality of less successful variants relative to more successful ones, gradually gaining resistance in a step-wise fashion. Whether or not the cells can gain resistance fast enough before succumbing to the therapy determines whether they will remain extant or go extinct.

### Self-genetic modification

**Figure 2:**
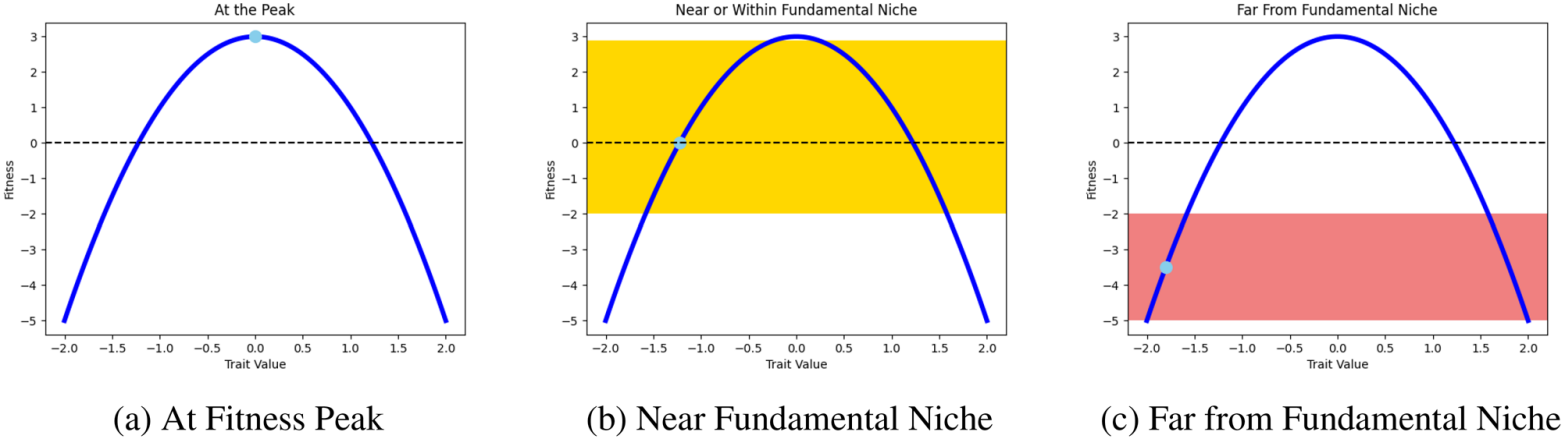
When evolutionary triage and self-genetic modification occurs. The curves depicted here are sample, schematic adaptive landscapes. They are not representative of any underlying biological phenomenon, but are rather provided as an illustration of what happens when species are at, close to, or far from an evolutionary peak. When cells are at a fitness peak (**a**), technically, due to a lack of selection pressure, no evolution occurs. When cells are bobbing near the sea level (**b**), only evolutionary triage occurs. When cells are drowning (**c**), primarily self-genetic modification occurs, though evolutionary triage can still occur via the 2N+ state.

In SGM, as in the case of ET, an environmental stressor promotes the transition of cells from the 2N+ state into the PACC state. However, what happens next depends on how extreme this stressor is or, more accurately, what the fitness and internal condition of the cell is. If cells are near or within their fundamental niche (either above or slightly below “sea level”)^1,85^, evolutionary triage will occur, just as described above. However, if cells are “drowning”, far from their fundamental niche, the SGM program will commence. Namely, cells in the PACC state will assess lack of viability, remain in this state until they find a sufficient solution, and then resume proliferation and evolution via ET. There are two main components to this: generating and assessing a solution. Cells may generate solutions via a “single-shot” approach: a mutation is produced and, if it’s sufficient, the mutation is kept, a mutant progeny with this trait is produced, and proliferation resumes. If it’s not sufficient, the mutant trait is discarded and the process repeats. However, we assume that cells generate solutions via a gradualistic approach that parallels ET. In this process, a mutant trait is produced. If the fitness of cells with this trait would be higher than that with the current trait, the mutant trait will be adopted and evolution will build upon this. If it is not better than the resident trait, it will be discarded. It is critical to note that in this process, 2N+ progeny *will not* be produced by cells in the PACC state until a sufficient solution is reached. Although the mechanism by which the assessment of solutions is unknown, we propose a conceptual framework to help us crudely understand how this happens. When cells in the PACC state produce a mutant trait under SGM, a mutant progeny is not produced. Rather, via an internal process of trial-and-error, a “hypothetical progeny” is produced and tested to examine how viable it would be in the harsh external environment. If it would be more viable than a progeny with the current trait, the PACC will adopt this mutant trait as its new baseline and continue this process. Theoretically, an SGM threshold controls just how viable this progeny needs to actually be produced by the PACC. When this threshold is met, cells in the PACC state can undergo depolyploidization into the 2N+ state with this trait and can continue proliferating and evolving via ET. An outline of how the ET and SGM hypotheses were simulated can be seen in Fig. 1. To more clearly grasp when the phases of evolutionary triage and self-genetic modification occur in the SGM hypothesis, consider Fig. 2.

In addition to considering how the fitness of a cancer cell population influences the mode of evolution, it is critical to understand how this fitness is achieved. Namely, what conditions can cause an organism to be pushed outside its fundamental niche? We separate these factors into two groups: density-dependent and density-independent factors. Density-dependent effects refer to any process in which the fitness of an organism is regulated by the density of the population, e.g., via competition for resources. Density-independent factors on the other hand, impact an individual’s fitness regardless of population density, e.g., therapy (viewed simplistically).

To distinguish between these two forms of fitness regulation, we introduce the concept of soft and hard selection regimes. Under soft selection, ecological disturbances may greatly reduce environmental quality, thereby reducing the carrying capacity and supporting fewer cells. However, there is still a positive ecological equilibrium under the disturbance. As the population size decreases, the fitness of extant cells increases dramatically and some cells are able to persist in the population. Although evolution may help increase population size in this case, density dependent factors play a much stronger role at regulating the fitness of the cells. Under hard selection, ecological catastrophes do not permit a positive ecological equilibrium. Under this scenario, evolution is critical since the current strategy cannot maintain a population. Although a reduction in population size may increase the fitness of the remaining cells, this is not sufficient to prevent extinction. In this paper, we assume that SGM comes into play when cells are under hard selection regimes and when quickly evolving a viable strategy is essential for cells to persist. As we will soon see, we enforce this by using the effect of therapy as a marker for when SGM is implemented.

## Results

Using this framework, we examined how drug dosage and extent of self-genetic modification affect the population and resistance strategy dynamics of cancer cell populations under ET and SGM. To supplement these investigations, we ran *in silico* competition experiments by competing cancer populations under ET with populations under SGM under soft (low drug dosage) and hard (high drug dosage) selection regimes. In all our simulations, we ran 100 trials for robustness. We hypothesized that higher doses of drug will favor SGM as it allows cancer cells to persist in a stable PACC state until they find a viable solution to the stressor. Conversely, we expected ET to perform better under low doses of therapy due to density-dependent competition factors that become more critical, as the importance shifts away from simply surviving the stressor to also competing with other cancer cells. We anticipated that the higher the degree of self-genetic modification, the longer the cancer cell population will remain in the stable PACC state.

### Cancer eco-evolutionary dynamics

**Figure 3:**
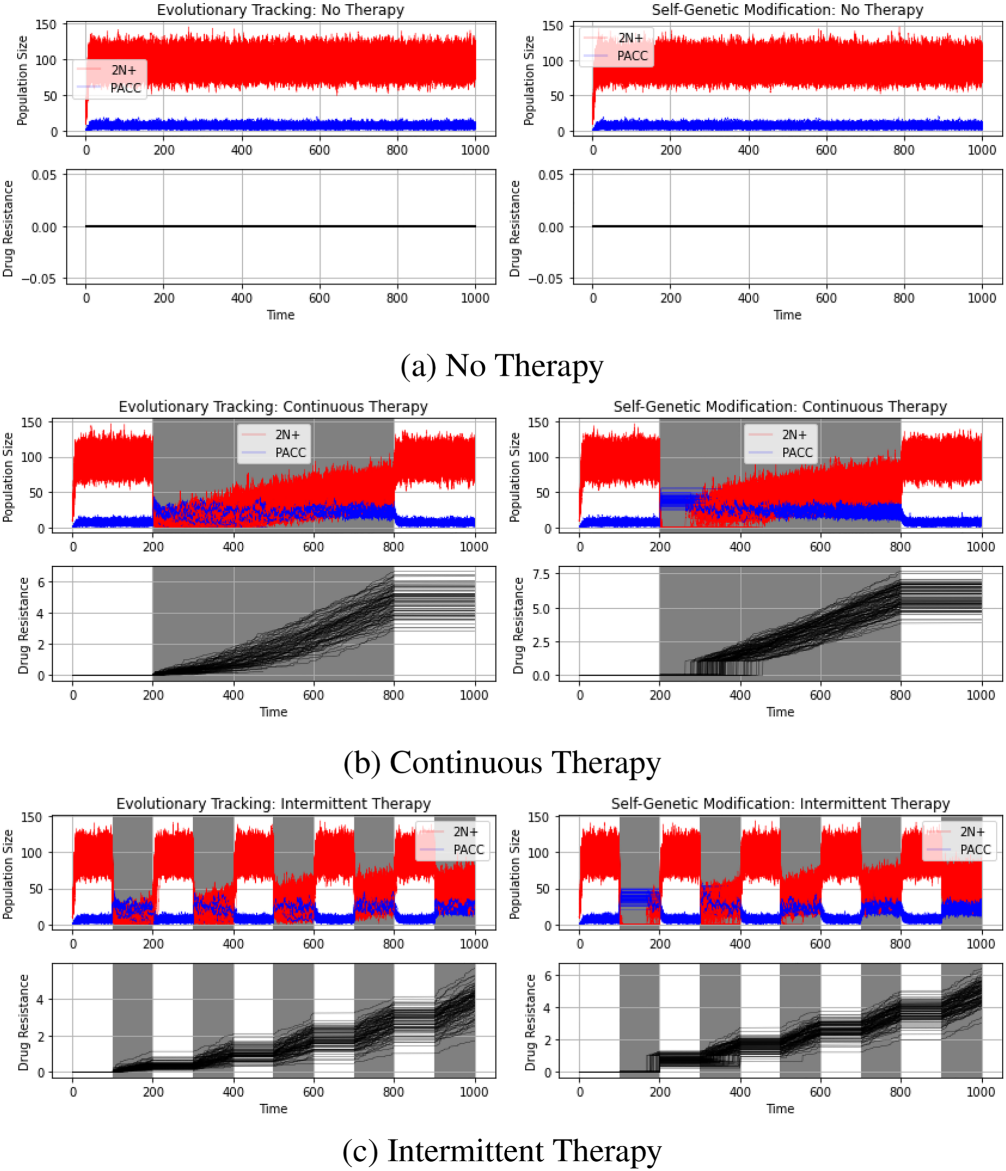
No therapy (**a**), continuous therapy (**b**), and intermittent therapy (**c**) under evolutionary triage and self-genetic modification. Under SGM, the stable PACC state allows cells to persist under therapy and buys them enough time to evolve adequate levels of resistance and avoid extinction. Under ET, in most cases, the cells are able to evolve resistance quickly enough to avoid extinction; however, extinction still occurs in a minority of cases.

First, we simulated basic dynamics of a cancer cell population with 2N+ and PACC states using the technique outlined above under evolutionary triage and self-genetic modification. Specifically, we analyzed population and strategy dynamics under the cases of no therapy, continuous therapy, and intermittent therapy. Each line in all the following stochastic simulations corresponds to the results of one simulation. For continuous therapy, the drug was administered from time 200 to 800. Under intermittent therapy, the drug was administered for 100 time steps, followed by a drug holiday for 100 time steps, repeated over the duration of the simulation. In both cases, we used a drug dosage of *m* = 0.8 and a self-genetic modification threshold of $\zeta =0.4$. For all simulations in this subsection, the cancer cell population was initialized with 10 cells in the 2N+ state, 0 cells in the PACC state, and with a resistance strategy of 0. The results from these simulations can be seen in Fig. 3.

In each of the following simulations, the top panel captures the population dynamics of cancer cells in the 2N+ (red curves) and PACC (blue curves) states. The bottom panel captures the evolutionary dynamics of resistance (black lines). The regions shaded in gray depict the intervals during which therapy is administered; unshaded regions are those during which no therapy is given.

First, consider the control case of no therapy (Fig. 3a). As we expect, there is no evolution in the drug resistance trait (as no selection pressure has been induced) and the population quickly equilibrates to predominantly cells in the 2N+ state. No population goes extinct and the eco-evolutionary dynamics under ET and SGM are virtually identical. Next, let’s consider the case of continuous therapy (Fig. 3b). Under ET, 27% of populations went extinct, whereas none went extinct under SGM. In both cases, there is a drastic shift of cells from the 2N+ state to the PACC state upon induction of therapy. As cells gain resistance, the need for great heritable variation diminishes. This leads to a gradual decrease of cells in the PACC state, accompanied by a corresponding increase of cells in the 2N+ state. Under SGM, as soon as therapy is administered, cells enter a stable PACC state that lasts about 100 time steps. This gives the cancer cells time to achieve adequate levels of resistance. After this, cells in the PACC state begin to undergo depolyploidization and evolutionary triage commences. This trend is paralleled in the evolutionary dynamics of resistance, where we see a period of evolutionary stasis until around time 300, at which point we see rapid evolutionary change followed by more gradualistic evolution until therapy is removed. Similar trends can be seen in the intermittent therapy simulations (Fig. 3c), in which 13% of ET populations and 0% of SGM populations went extinct. During periods of treatment, the frequency of cells in the PACC state increases and the frequency of cells in the 2N+ state decreases. However, as resistance emerges, a lower frequency of cells in the PACC state is noticed during times of treatment, in accordance with the reduced need for great heritable variation. In the SGM case, we note that the stable PACC state lasts for the first treatment cycle and ET begins soon after the second cycle begins.

**Figure 4:**
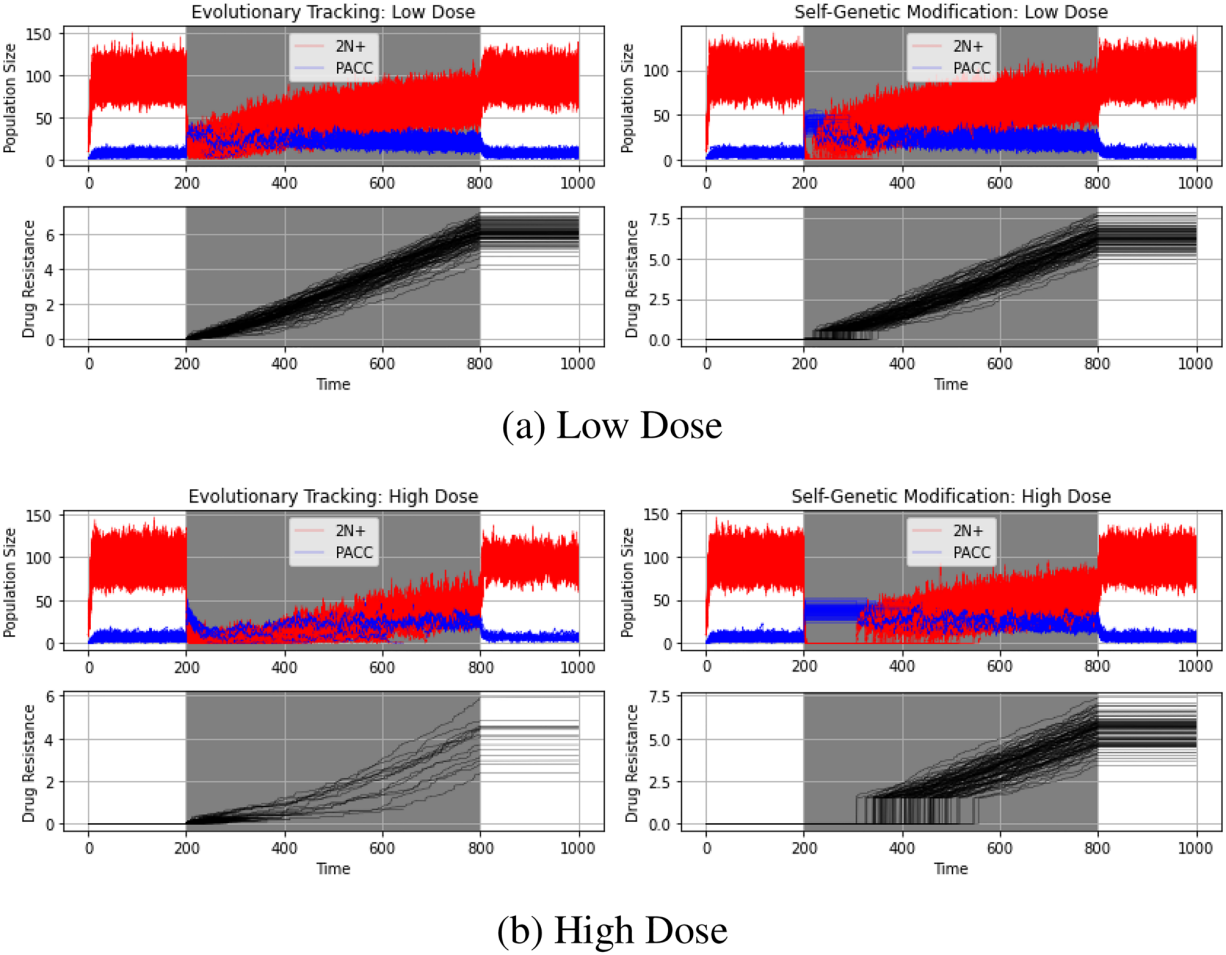
Low dose (**a**) and high dose (**b**) continuous therapy under evolutionary triage and self-genetic modification. The higher the dose of therapy, the longer cells remain in the stable PACC state under SGM. Under ET, all populations avoid extinction under low dose therapy, but the vast majority go extinct when exposed to a high dose of therapy.

Next, we examined how different drug doses impact the eco-evolutionary dynamics of cancer cell populations under ET and SGM. We expected that higher doses of drugs will result in a higher extinction rate and a longer stable PACC state. To test our intuitions, we simulated population and resistance strategy dynamics under low dose (*m* = 0.6) and high dose (*m* = 1) therapy that is continuously administered from time step 200 to 800. The results of these simulations can be seen in Fig. 4.

First, let’s consider the low dose simulation results (Fig. 4a). In this case, none of the ET or SGM populations go extinct. The similarity of the eco-evolutionary dynamics under the ET and SGM is due to the shorter stable PACC state. Since a low dose of therapy was given, death due to drug is lower and it takes less time for the cancer cell population to evolve an adequate resistance strategy. Now, let’s shift our focus to the high dose simulation results (Fig. 4b). In this case, 85% of ET populations went extinct, whereas all SGM populations avoided extinction. Note that it is the immediate transition to the stable PACC state that allows cancer cells under SGM to remain extant in the population while evolving resistance. When exposed to extreme environmental conditions such as a high dose of therapy, this stable PACC state exists for longer, giving the cancer cell population enough time to evolve resistance and effect evolutionary rescue^86^. On the other hand, ET populations cannot access this mechanism. As a result, cells in the 2N+ state often go extinct, followed shortly after by the eradication of cells in the PACC state.

**Figure 5:**
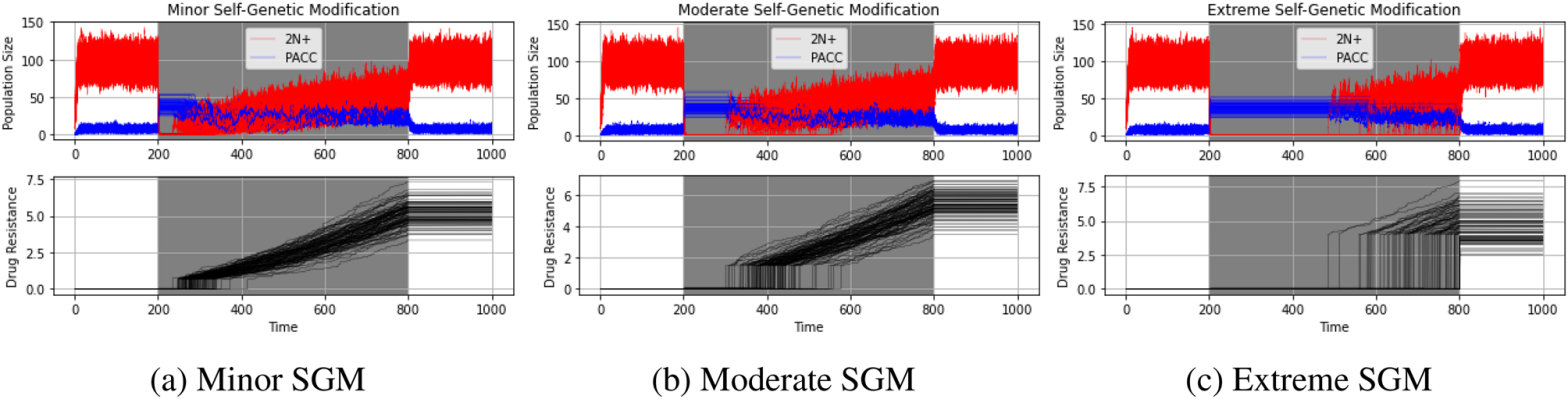
Population and strategy dynamics for self-genetic modification under continuous therapy. The degree of self-genetic modification influences when the cancer cell population shifts from self-genetic modification to evolutionary triage: the greater the degree of self-genetic modification, the longer the cells will remain in a stable PACC state.

In the SGM simulations thus far, we assumed that cells in the PACC state undergo depolyploidization into the 2N+ state only when drug kill is less than 0.4. To explore how the extent of self-genetic modification impacts eco-evolutionary dynamics of cancer cell populations under SGM, we compared extreme ($\zeta =0.2$), moderate ($\zeta =0.4$), and minor ($\zeta =0.6$) SGM. Note that when $\zeta =1$, the SGM hypothesis is analogous to ET and when $\zeta =0$, the SGM hypothesis functionally serves as a “hibernation state” that cancer cells enter every time they are exposed to therapy. To clearly show the differences among these levels of self-genetic modification, we used a high dose therapy (*m* = 1) that is administered continuously from time step 200 to 800. The results of these simulations can be seen in Fig. 5.

As expected, none of the populations go extinct in any of the simulations, regardless of the degree of SGM. We note that the more extreme the degree of SGM, the longer the cells remain in their stable PACC state. This is clearly paralleled in the evolutionary resistance strategy dynamics, in which we see a longer period of evolutionary stasis and a greater burst of evolutionary change for more extreme SGM mechanisms.

### In silico competition experiments

**Figure 6:**
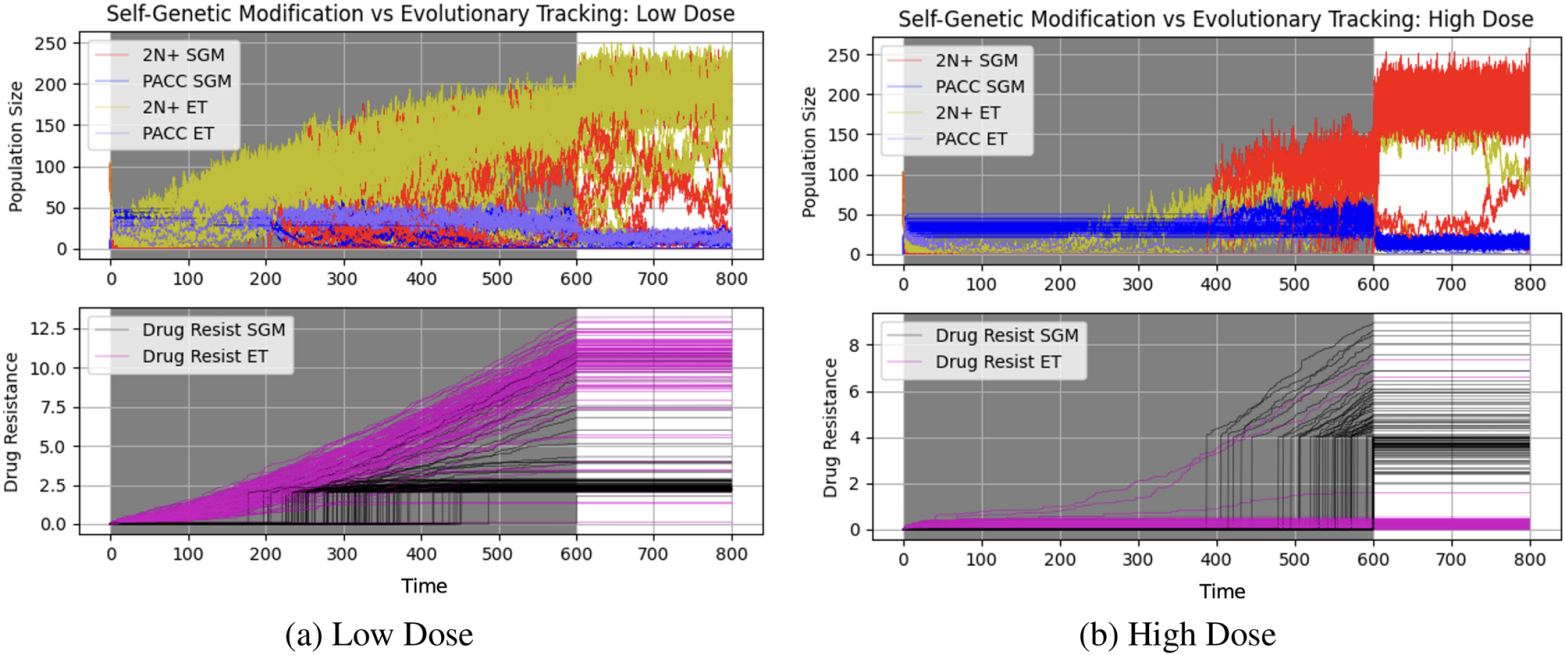
Competitive dynamics of cancer populations with self-genetic modification and evolutionary triage under continuous therapy. Under low dose therapy (**a**), cell population under ET outcompete those under SGM in the majority of cases. Conversely, under high dose therapy (**b**), cell populations under SGM outcompete those under ET.

From the simulations thus far, it seems that SGM is favored over ET in nearly all cases. However, since we simulated the ET and SGM populations independently, the density-dependent factors between these populations were inherently ignored. This competition becomes particularly important under soft selection regimes in which external selection pressures are not significant. ET allows cells to continue proliferating at all times, unlike SGM that halts growth of the population during the stable PACC phase. Thus, we hypothesized that when cells are under low stress environments, ET will be favored due to its density-dependent competitive advantage. Conversely, when cells are under high stress environments and must undergo evolutionary rescue to avoid extinction, SGM will be favored since cells under ET will not be able to evolve fast enough to persist in harsh conditions without access to the PACC state. When cells are in an environment with no or very little stress, the SGM and ET hypotheses are identical and thus, both will do equally well. Here, we competed cancer cell populations under ET and SGM against each other under low (*m* = 0.6) and high (*m* = 1) dose continuous therapy. To clearly note the difference between ET and SGM, we used a self-genetic modification threshold of $\zeta =0.2$. To avoid initial stochastic effects that can cause extinction of either population before therapy is administered, we gave therapy from time 0 to 600 and ran the simulation for 800 time steps. We initialized the cancer cell populations with 100 cells in the 2N+ state, 0 cells in the PACC state, and resistance strategies of 0 for both the ET and SGM populations. The results of these simulations can be seen in Fig. 6.

**Figure 7:**
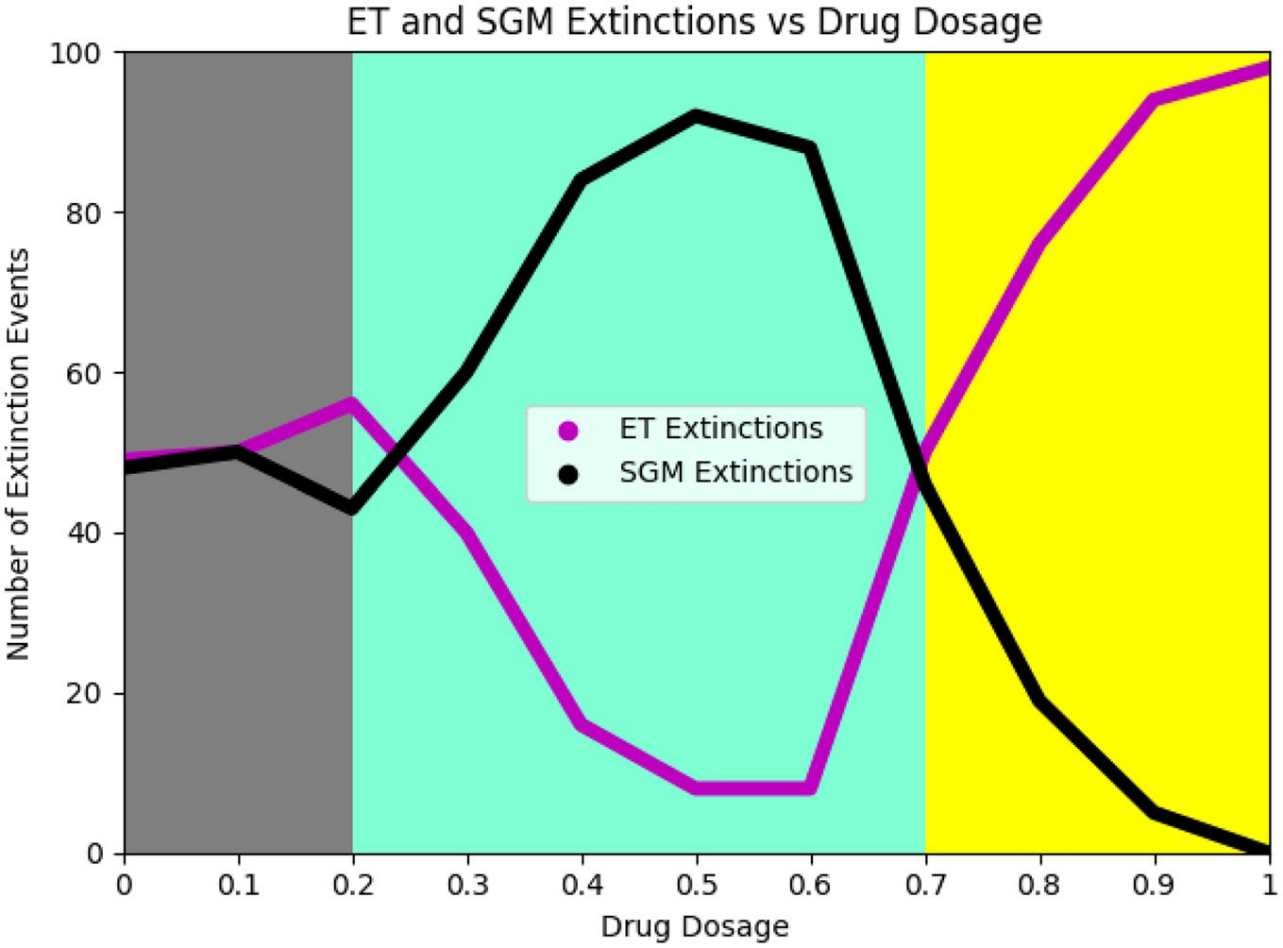
Extinction rates for evolutionary triage and self-genetic modification for various drug doses. ET is favored under low selection regimes and SGM is favored under high selection regimes. When external selection pressures are very low, SGM and ET mechanistically act in an identical fashion.

In these plots, the red and blue population curves represent cells in the 2N+ and PACC states under SGM. The yellow and purple population curves represent cells in the 2N+ and PACC states under ET. The black and magenta curves in the bottom panel capture the evolutionary resistance dynamics of SGM and ET populations, respectively. As we hypothesized, under the low dose treatment regimen (Fig. 6a), 88% of the SGM populations went extinct as compared to only 8% of the ET populations. Conversely, under high dose therapy (Fig. 6b), 98% of the ET populations went extinct, whereas none of the SGM populations went extinct. To more finely explore the interplay between drug dosage and ET/SGM evolutionary mechanisms, we run simulations competing these populations against each other for drug doses between 0 and 1 (at intervals of 0.1). A summary of the results of these simulations can be seen in Fig. 7.

In this plot, we depicted the number of extinction events (out of 100) for ET and SGM populations competed against each other for a range of drug doses. The magenta curve captures ET extinctions and the black curve captures SGM extinctions. The region shaded in gray is the “no selection” regime, in which the ET and SGM mechanisms are identical. The aqua shaded portion is the “low selection” regime, where the ET mechanism is favored. The yellow shaded part is the “high selection” regime, in which SGM performs better. As per our hypothesis, evolutionary triage seems to achieve an advantage through density-dependent competition under the low selection regime. However, in the high selection regime, the importance of density-dependent factors is overshadowed by the necessity to simply survive in the face of extreme stressors. The stable PACC state afforded by self-genetic modification gives it this advantage.

## Conclusion

Therapeutic resistance is a major cause of treatment failure in patients with advanced cancers. We created a stochastic model to examine two potential mechanisms by which a key contributor to therapeutic resistance, the PACC state, allows cancer cells to persist and adapt to extreme environmental stressors: evolutionary triage and self-genetic modification. In doing so, we provided a general framework that allows us to model mechanisms of reverse transcriptional inheritance and compare them to traditional Mendelian inheritance. We used this framework to simulate the eco-evolutionary dynamics of cancer cell populations under therapy. We investigated how these dynamics change due to drug dosage and extent of self-genetic modification. Finally, we supplemented these studies with *in silico* competition experiments where we competed cancer cell populations under evolutionary triage and under self-genetic modification against each other under low and high dose therapeutic regimens. Most importantly, we found that self-genetic modification is favored under extreme stressors, in which simply surviving is more important than density-dependent competition. Conversely, evolutionary triage performs better under more modest stressors, as the importance of density-dependent competition comes to the fore.

These findings have broad implications for both cancer biology and evolutionary biology. Our results provide qualitative predictions for how the PACC state contributes to therapeutic resistance through evolutionary triage and self-genetic modification. These results can help guide future experimental work to empirically examine how SGM and ET work together to contribute to resistance. Our work also provides a generalizable framework within which hypotheses and mechanisms of Lamarckian and Mendelian inheritance can be simulated and compared. Lamarckian inheritance is often erroneously conceptualized as antithetical to classical evolution by natural selection. However, in our work, we show how it can be naturally placed in a Darwinian framework. Though we focus on cancer here, we expect that these findings are broadly applicable to any system capable of evolving.

## Supplementary Information

Supplementary Information.

## Data Availability

The datasets generated during and/or analysed during the current study are available in the Self-Genetic Modification repository in Anuraag Bukkuri’s GitHub, at https://github.com/abukkuri/Self-Genetic-Modification/.
