# Supplementary material for: Stochastic models of Mendelian and reverse transcriptional inheritance in state-structured cancer populations
Source: Sci Rep. 2022 Jul 29;12:13079. doi: 10.1038/s41598-022-17456-w (PMC9338039; doi:10.1038/s41598-022-17456-w)
Supplement: Supplementary file 1 — Supplementary Information. [file 41598_2022_17456_MOESM1_ESM.pdf]

Supplementary Material

Anuraag Bukkuri[1,*], Kenneth J. Pienta[2], Robert H. Austin[3], Emma U. Hammarlund[4], Sarah R. Amend[2], and Joel S. Brown[1]

[1]Cancer Biology and Evolution Program and Department of Integrated Mathematical Oncology, Moffitt Cancer Center
[2]The Brady Urological Institute, Johns Hopkins School of Medicine
[3]Department of Physics, Princeton University
[4]Nordic Center for Earth Evolution, University of Southern Denmark and Translational Cancer Research, Department of Laboratory Medicine, Lund University

July 19, 2022

1 Birth-Death-Switching Process Algorithm

Here, we present a way to mathematically formalize and simulate the intuitive explanation given for the evolutionary dynamics in Section 2.2. For a clear outline of this process, please refer to Figure 1 in the main text that provide flowcharts for how the evolutionary triage and self-genetic modification hypotheses were simulated.

We begin by computing birth, death, and switching (or transition) rates from Equation 1 at each time step. We then calculate the total event rate by summing the birth, death, and switching rates. We use this event rate to determine the time to the next event by sampling from an exponential probability distribution with a mean of the event rate. Next, we choose an event type based on the relative contributions of each type to the total event rate. We then carry out this event and repeat the process. If the event type is death, we eliminate one cell (since we assume cells in the PACC state do not die, the eliminated cell will always be in the 2N+ state). If the event type is switching from 2N+ to PACC, we eliminate one cell in the 2N+ state and produce one cell in the PACC state with a probability η to account for unsuccessful polyaneuploid transition due to mitotic catastrophe.

The other two events, birth (2N+ division) and switching from PACC to 2N+ (depolyploidization) require more thought. For these events, there is a chance (μ) for a mutation to occur. We sample the additive value of the mutation from a Gaussian distribution with mean 0 and breadth σ_i where $\sigma_1 < \sigma_2$ represent the breadths for division and depolyploidization events, respectively. Assuming invasion implies fixation [1, 2], if the mutation confers a higher fitness, it will replace the current trait value in the population. If the event is a birth and if no mutation occurs or the mutation confers a higher fitness, one cell in the 2N+ state will be added to the population. Now, let's consider depolyploidization switching events. The ecology of how the population size changes in this case depends on whether we are operating under the ET or SGM

hypothesis and, for the latter, the nature of the stressor. Under the ET hypothesis, if no mutation occurs or the mutation confers a higher fitness, we eliminate one cell in the PACC state and generate two cells in the 2N+ state. Under the SGM hypothesis, what occurs depends on the fitness of the cells, namely whether they are near/within their fundamental niche or far from it. In our case, this is determined entirely by the death due to drug term. To distinguish between these two regimes, we introduce an SGM threshold, ζ. If drug kill exceeds this term, the cells will operate under SGM; if it is less than ζ, cells will operate under ET. If $\zeta = 0$, SGM is identical to ET. If $\zeta = 1$, SGM simply serves as a state of resistance that cells always enter during therapy, despite their level of adaptation to the stressor. If cells are near or within their fundamental niche, a process identical to that under ET occurs. However, if cells are far outside their fundamental niche, the SGM program commences. Behind the scenes, cells in the PACC state continue evolving in a gradualistic fashion as in the ET case. However, until the death due to drug term is less than ζ, cells in the PACC state will not undergo deplyploidization, i.e., no change will occur in the 2N+ or PACC populations. Once the cells have identified a strategy that is good enough (i.e., would result in a drug kill term less than the SGM threshold), depolyploidization can occur: one cell in the PACC state is eliminated and two cells in the 2N+ state are generated. Importantly, note that under SGM, evolution can occur without the death of less fit variants relative to more fit ones. After the SGM phase, the population will be near or within their fundamental niche, so ET can commence. Under this hypothesis, it is worth stressing the non-exclusivity of SGM and ET [3]: it is possible and perhaps likely that some organisms evolve via SGM under high stress conditions and evolve via ET under more moderate stressors.

It is critical to stress that our stochastic simulation is not an agent-based model, but rather implements the stochastic element at a population level. We do not carry out events on specific, individual cells. Instead, we endow all cells within a given state with the same trait values and vital rates. Since cells do not have individual properties under this formulation, events merely occur to one cell in the state that is chosen, and we track changes in population size and trait value in each of the states.

2 Sensitivity Analysis

Here, we provide a brief sensitivity analysis of our stochastic model for key parameters. To do this, we run 100 trials of our simulated model using a range of values for each parameter we consider and plot the extinction rates. For simplicity, we hold all other parameters in our model constant and administer a continuous dose of therapy (with $m = 0.8$) between times 200 and 800. The summarized results can be seen in the figure below.

First, we consider the impact of the intrinsic growth rate, r, on extinction rates in Figure 1a. For low values of r, the cancer cell population is not able to divide fast enough to avoid extinction due to therapy. We notice a sharp decrease in extinction rates for $r \in [0.4, 0.7]$, after which virtually no extinctions are noticed. The cells are able to divide and evolve resistance fast enough to remain extant. Next, we consider the role of the obligate transition rate in Figure 1b. As we can see, as γ increases, the extinction rate decreases gradually. This is because the higher level of baseline PACCs allows the cells to survive the initial precipitous drop in population size upon the application of therapy. Similarly, we notice the extinction rate decrease as the resistance strategy efficacy increases in Figure 1c. This is because, as b increases, the effects of therapy are more quickly minimized by the evolution of resistance. Now, we consider the impact of facultative transitions to the PACC state in Figure 1d. For low values of c_{21}, extinction remains near 100: the cells are not able to transition into the PACC state fast enough to seek refuge from therapy and evolve an adequate resistance strategy. Once $c_{21} > 0.3$, the extinction rate gradually decreases as the facultative transition rate increases. For similar reasons, we notice the opposite trend in Figure 1e when

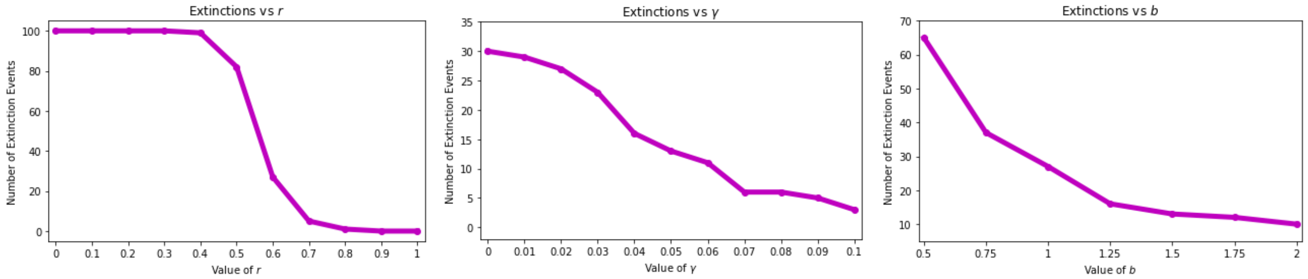

(a) Intrinsic Growth Rate (b) Obligate Transition Rate (c) Resistance Strategy Efficacy

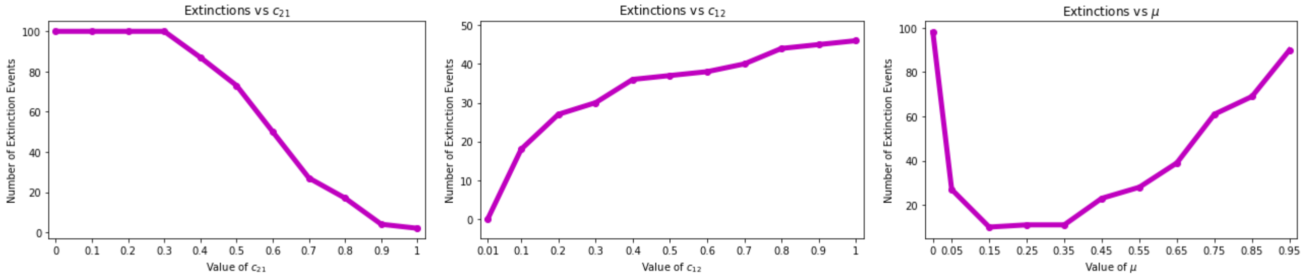

(d) Facultative Transition Scaling (e) Depolyploidization Rate (f) Mutation Rate

Supplementary Figure 1: Sensitivity Analysis of Model to Key Parameters.

we consider the effects of depolyploidization rate: the faster cells in the PACC state transition into the 2N+ state, the greater the extinction rate is. Finally, consider the effect of mutation rate on extinction in Figure 1f. For mutation levels lower than 0.05, high levels of extinction are noticed. Mutations fuel the evolution of resistance, so the lack of mutations hinders resistance from emerging, promoting extinction. For $\mu \in [0.05, 0.45]$, we notice a low level of extinctions. However, when we increase mutations past this, extinction rates increase dramatically. This is because too many mutations causes cell divisions to fail at an alarmingly high rate, leading to the extinction of the cancer cell population, often even before the administration of therapy.

References

[1] O Diekmann. A Beginner's Guide to Adaptive Dynamics. *Mathematical Modeling of Population Dynamics*, 63:47–86, 2004.

[2] S. A.H. Geritz, M. Gyllenberg, F. J.A. Jacobs, and K. Parvinen. Invasion dynamics and attractor inheritance. *Journal of mathematical biology*, 44(6):548–560, 6 2002.

[3] Eugene V. Koonin and Yuri I. Wolf. Is evolution Darwinian or/and Lamarckian? *Biology Direct*, 4:42, 2009.
